# Fulminant myocarditis associated with severe fever with thrombocytopenia syndrome: a case report

**DOI:** 10.1186/s12879-019-3904-8

**Published:** 2019-03-18

**Authors:** Shotaro Miyamoto, Takashi Ito, Shinsaku Terada, Tomohiro Eguchi, Hiroaki Furubeppu, Hideki Kawamura, Tomotsugu Yasuda, Yasuyuki Kakihana

**Affiliations:** 1 Emergency and Intensive Care Medicine, Kagoshima University Graduate School of Medical and Dental Sciences, 8-35-1 Sakuragaoka, Kagoshima, 890-8544 Japan; 2 Department of Infection Control and Prevention, Kagoshima University Hospital, 8-35-1 Sakuragaoka, Kagoshima, 890-8544 Japan; 3 Division of Intensive Care Medicine, Kagoshima University Hospital, 8-35-1 Sakuragaoka, Kagoshima, 890-8544 Japan

**Keywords:** Severe fever with thrombocytopenia syndrome, Fulminant myocarditis, Real-time RT-PCR

## Abstract

**Background:**

Severe fever with thrombocytopenia syndrome (SFTS) is an emerging viral infectious disease with high mortality. It causes multiple organ dysfunction; however, myocarditis has never been reported as a complication with SFTS.

**Case presentation:**

A 62-year-old previously healthy woman developed fever, fatigue, diarrhea, and a mild consciousness disorder. She visited a local clinic, and laboratory data showed leukocytopenia, thrombocytopenia, and elevation of the aspartate aminotransferase level. She was transferred to Kagoshima University Hospital and diagnosed as having SFTS by real-time reverse transcription polymerase chain reaction. Subsequently, her blood pressure gradually decreased despite fluid resuscitation and vasopressor administration. Based on elevated toroponin I levels in serum, a transient diffuse left ventricular hypokinesis and wall thickening in echocardiography, diffuse ST elevation in electrocardiography, and exclusion of other heart diseases, she was diagnosed as having fulminant myocarditis. After hemodynamic support with inotropic agents, she recovered near normal cardiac function. She was discharged to home on day 28.

**Conclusions:**

We report the first case of fulminant myocarditis associated with SFTS.

## Background

Severe fever with thrombocytopenia syndrome (SFTS), a form of viral hemorrhagic fever (VHF), is an emerging infectious disease with high mortality [1]. It was first reported in China in 2011 [1] and subsequently in Korea [2] and Japan [3]. SFTS is caused by the SFTS virus (SFTSV), which belongs to the genus Phlebovirus of the family Bunyaviridae [2]. SFTSV is transmitted to humans through tick bites [2], human blood [4], bodily fluids [5], and probably aerosols [6, 7]. The diagnosis of SFTSV infection has been generally made base on the detection of the SFTSV ribonucleic acid (RNA) in serum by real-time reverse transcription polymerase chain reaction (RT-PCR) [2, 8], and the number of SFTS cases has increased recently [9]. In china, 7419 cases of SFTS were reported 2010–2016 [10], with the case-fatality rate ranged from 6.4 to 12.2% [11, 12]. In Japan, 397 cases of SFTS have been reported as of January 30, 2019; 65 cases (16%) were fatal at notification time (National Institute of Infectious Diseases). The typical clinical symptoms of SFTS include hemorrhagic fever, gastrointestinal problems, thrombocytopenia, and leukocytopenia [2].

In addition, central nervous system symptoms, renal and hepatic failure, rhabdomyolysis, and hemophagocytic syndrome have been common in SFTS patients [13]. Further, severe cases could be complicated by immune disturbance, as illustrated by cytokine storm and T cell immunodeficiency, leading to fatal outcome [14, 15]. Thus, although SFTS is characterized by multiple organ failure, no studies have been reported of fulminant myocarditis with SFTS.

Here, we present an atypical SFTS case in which the patient experienced cardiogenic shock because of fulminant myocarditis but whose subsequent course was nevertheless good.

## Case presentation

A previously healthy 62-year-old woman living in a rural area developed fever, headache, and fatigue starting on July 27, 2018. She also had gastrointestinal symptoms, such as anorexia, nausea, and diarrhea later, and visited a local clinic on July 30. She was prescribed antipyretic analgesics and returned home. The following day, she developed a mild consciousness disorder and visited a local clinic again accompanied by her family. She underwent a magnetic resonance imaging examination, but no abnormality was noted. However, laboratory data showed leukocytopenia, thrombocytopenia, and increased aspartate aminotransferase (AST) and creatine kinase (CK) levels. She was transported to Kagoshima University Hospital.

On admission, she had a slight disturbance of consciousness [Glasgow Coma Scale of 14: E3, V5, M6], a body temperature of 37.4 °C, a respiratory rate of 22/min, blood pressure of 109/73 mmHg, heart rate of 97/min, SpO_2_ of 99% (room air), normal heart and respiratory sound, no swelling of the superficial lymph nodes, crusty bite wounds in the left inguinal region, although there was no tick. Laboratory testing showed leukocytopenia (total white blood cell count of 910 cells/mm^3^); thrombocytopenia (platelet count of 63,000 cells/mm^3^); mildly elevated AST (68 U/L), lactate dehydrogenase (364 IU/L), and CK (317 U/L) levels; elevated ferritin level (1947 ng/mL); and normal CK-MB fraction (CK-MB) (4 U/L) and C-reactive protein (CRP) level (< 0.02 mg/dL). A urinary general examination showed protein (4+) and occult blood (3+). Electrocardiography (ECG) and chest radiography findings were normal on the day of admission (day 1).

Because of these clinical findings, she was suspected of having SFTS and transferred to the intensive care unit. Several hours after admission, SFTSV RNA was detected in her serum samples by RT-PCR, so she was definitively diagnosed as having SFTS.

**Fig. 1:**
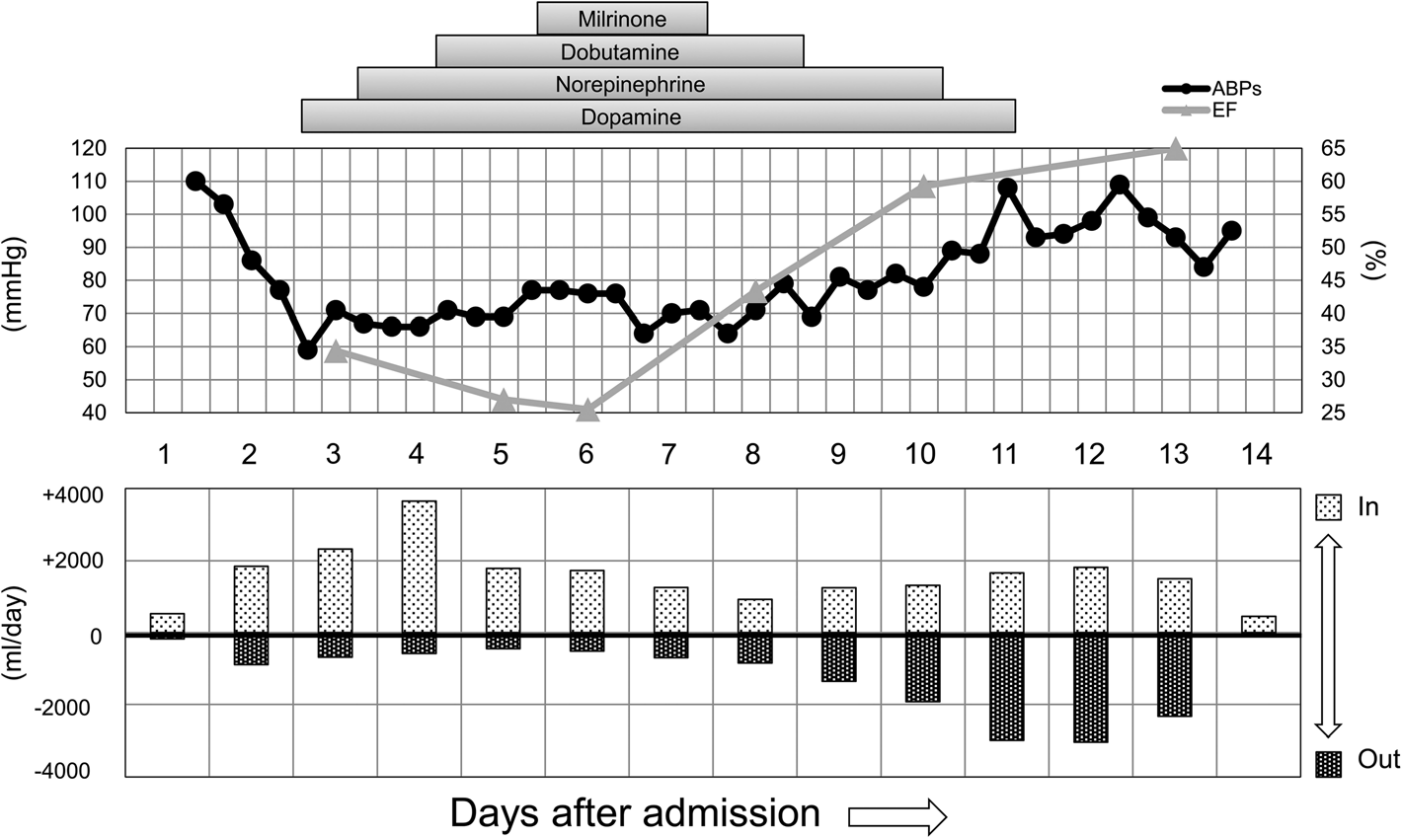
Clinical course during intensive care unit stay for 14 days. Changes of EF (M-Mode measurement of LV by Teichholz), ABPs (minimum per 8 h), in-out water balance per day. EF: ejection fraction; ABPs: systolic arterial blood pressure

**Fig. 2:**
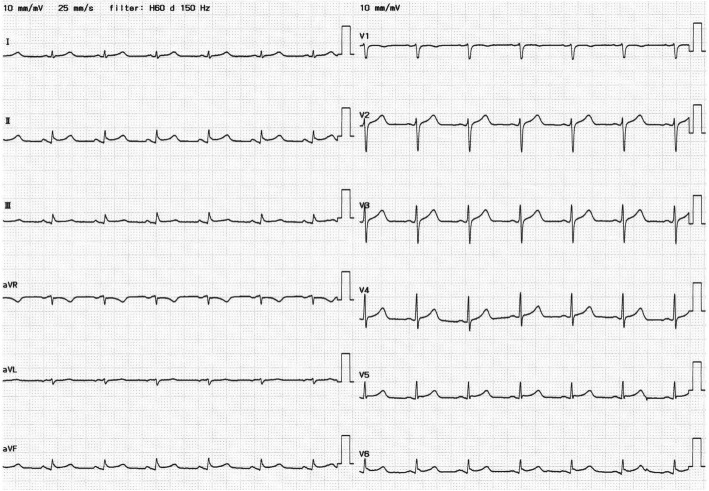
Electrocardiograms on day 3. ST-segment elevation in the II, III, aVf, and V2–6 leads and inverse T wave in aVr lead, and low voltage QRS complex (< 0.5 mV) in all leads

Her clinical course is shown in Fig. 1. Her blood pressure gradually started to decrease from day 2 evening. Fluid resuscitation (100 ml/h for 10 h; crystalloids 500 ml, colloids 500 ml) was performed until day 3 morning because hand-held ultrasound on day 2 midnight showed collapsed IVC (inferior vena cava). Then, repeat hand-held ultrasound showed recover from collapsed IVC; nevertheless, her blood pressure had not recovered and administration of low doses of dopamine and norepinephrine was started. To exclude cardiogenic shock, trans-thoracic echocardiography was performed and showed diffuse left ventricular (LV) wall motion depression (with an EF: ejection fraction of 34.4%), diffuse LV wall thickening, and small amount of pericardial effusion. Further, ECG showed ST elevation in the II, III, aVf, and V2–6 leads and a low-voltage complex in all leads (Fig. 2), and the level of troponin I was elevated (120.9 ng/mL), but that of CK-MB was not. She did not have any chest symptoms. She met several diagnostic criteria for clinically suspected myocarditis [16], which include unexplained cardiogenic shock, elevated level of troponin I, abnormal ECG and echocardiographic findings. In addition, she also met criteria for fulminant myocarditis [17], which include acute illness (history of < 2–4 weeks since the onset of symptoms), hemodynamic instability due to cardiogenic shock, and need for hemodynamic support (inotrope). Therefore, she was suspected of having fulminant myocarditis caused by SFTSV. However, we did not perform coronary angiography (CAG) or endomyocardial biopsy because thrombocytopenia and leukocytopenia greatly increased the risk of an invasive procedure. We screened for other viruses, including enterovirus, adenovirus, and influenza viruses that may cause myocarditis, but none of them were detected in nasal swab samples.

**Fig. 3:**
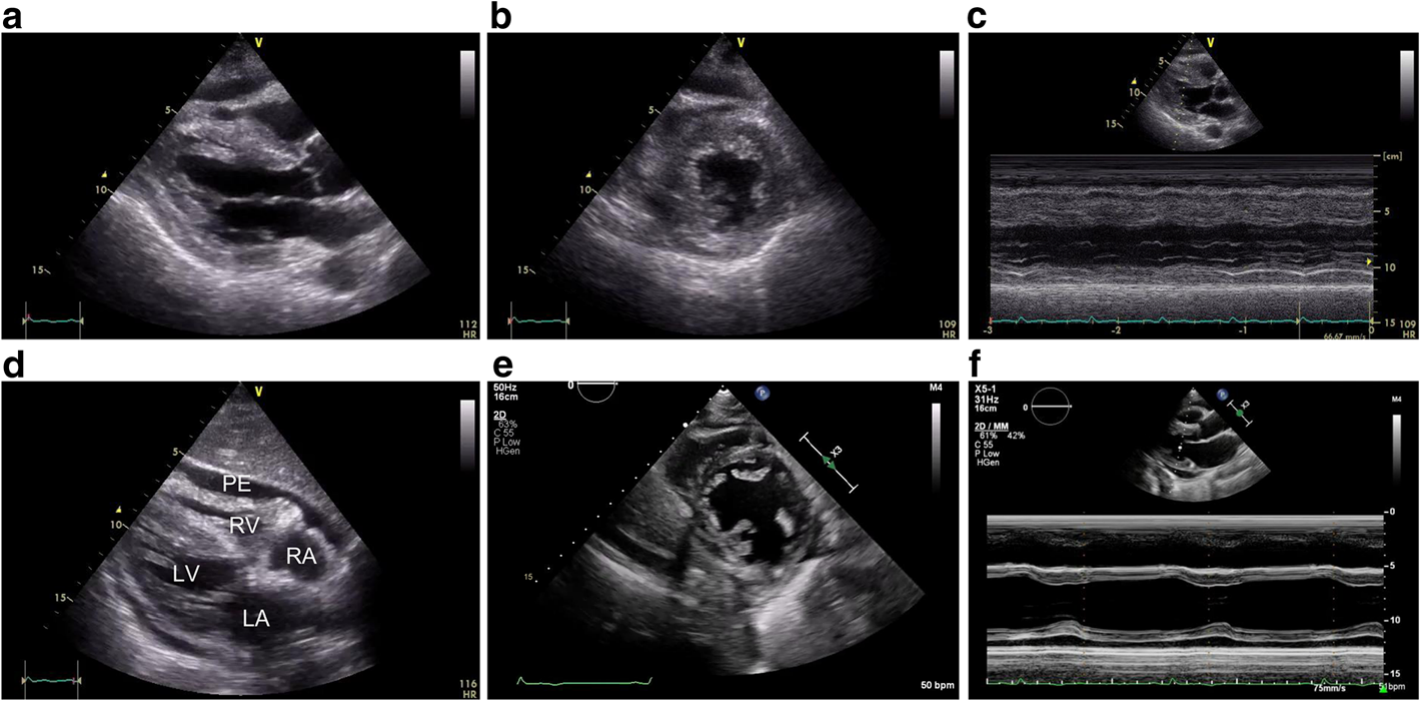
Echocardiograms on days 5 and 6, and 17. Day 5: Parasternal long- and short-axis end-diastolic views demonstrating thickening of the diffuse left ventricular wall and narrowing of the left ventricular cavity (**a**, **b**). M-mode Mesurement of left ventricle (LV) showing depressed LV contraction (**c).** Day 6: Subcostal four chamber view demonstrating small amount of pericardial effusion and right ventricular diastolic collapse (**d**). Day 17: Parasternal short-axis end-diastolic views demonstrating returned to normal wall thickness and cavity of LV (**e**). M-mode Mesurement of LV demonstrating normal LV contraction (**f**). PE: pericardial effusion; RA: right atrium; RV: right ventricle; LA: left atrium; LV: left ventricle

On day 5 of hospitalization, repeat echocardiography showed further LV dysfunction (with an EF of 27.9%), diffuse LV wall thickening (septum/posterior wall: 14.7 mm/13.5 mm), and narrowing of the left ventricular cavity (Fig. 3). On the basis of these results, administration of low doses of dobutamine and milrinone was added. On day 6 of hospitalization, repeat echocardiography showed cardiac tamponade findings of the dilated IVC without respiratory variations and right ventricular diastolic collapse (Fig. 3). However, no surgical pericardial puncture was performed, because there was no safe puncture space and a circulation agonist was able to maintain blood pressure. After that, echocardiography showed steady improvement of LV function (EF) and wall thickness, the urine output also continued to increase and the water balance gradually leaned toward negative as a result (Fig. 1). Additionally, the pericardial effusion gradually decreased and the cardiac tamponade findings naturally disappeared. On day 11 of hospitalization, her mild disturbance of consciousness from admission had improved, and she no longer needed a circulatory agonist. On day 13 of hospitalization, ECG showed improvement of ST elevation in the II, III, aVf, and V2–6 leads. She was eventually transferred to a general ward on the same day. On day 17 of hospitalization, repeat echocardiography showed normal LV function (with an EF of 70.3%) and wall thickness (septum/posterior wall: 8.9 mm/9.4 mm) (Fig. 3).

On day 20 of hospitalization, laboratory testing and ECG showed no abnormal findings. After exercise rehabilitation, she was discharged home on day 28 of hospitalization.

She had no history of cardiac diseases, allergy, or use of drugs that may cause myocarditis. During 28 days of hospitalization, CRP and bacterial culture were always negative, and there was no evidence of eosinophil elevation. Viruses, other than SFTSV, were not detected by virus isolation test. Based on these results, she was clinically diagnosed as having fulminant myocarditis caused by SFTSV.

## Discussion and Conclusions

According to the previous autopsy reports of SFTS patients [18, 19], SFTS nucleoprotein antigens have been found mainly in the lymph nodes, liver, spleen, and bone marrow by an immunostaining method; therefore, it has been presumed that lymphatic organs are the main sites of SFTSV proliferation. However, recent autopsy report of an SFTS patient without myocarditis demonstrated that by new immunohistochemistry and immunofluorescent staining methods, the least amount of SFTS nucleoprotein antigen was detected in the heart [20]. This suggested that SFTSV can directly infect the heart. Although severe SFTS cases could suffer myocardial dysfunction [13, 21], diffuse ST elevation in electrocardiogram or narrowing of LV cavity and diffuse LV wall thickness in echocardiogram were not evident in these cases, and thus these cases were not suspected of having myocarditis.

Myocarditis, which is known as inflammation of the myocardium [22, 23], is commonly caused by viral infection [24]. In the pathogenesis of viral myocarditis, the direct effect of the infecting virus on cardiomyocytes and secondary attack of immune cells against infected cardiomyocytes are important for myocardial damage [25]. These mechanisms lead to inflammation, necrosis, and apoptosis of cardiomyocytes [25]. However, because different viral infections cause the same outcome, immune response may play a more important role in the pathogenesis of myocarditis [26].

In the present case, the patient was diagnosed as having SFTS by RT-PCR 6 days after the onset of SFTS symptoms. Starting from the next day, the patient experienced circulatory collapse. Based on elevated troponin I levels in serum, diffuse LV wall thickening and hypokinesis in echocardiography, diffuse ST elevation in electrocardiography, the patient was suspected of having fulminant myocarditis. To definitively diagnose the patient as having fulminant myocarditis, endomyocardial biopsy is necessary. Endomyocardial biopsy is important not only for confirming diagnosis of myocarditis but also for diagnosing some specific types (giant cell, eosinophilic) of fulminant myocarditis that are considered to be effective for immunosuppressive therapy [27]. It is also important to perform CAG in patients suspected of having myocarditis to exclude acute myocardial infarction (AMI). However, endomyocardial biopsy cannot always detect the lesion, and because of the invasive procedure, there is a risk of arrhythmia and ventricular perforation [28]. In addition, the patient had thrombocytopenia and leukocytopenia. Therefore, we decided against performing endomyocardial biopsy and CAG and diagnosed her as having fulminant myocarditis clinically.

Table 1 shows that troponin I and CK continued to rise until day 7 and then declined with improvement in cardiac function, which seemingly reflected myocardial injury. However, CK-MB, which is a CK isozyme derived from cardiomyocytes, was always < 2% of CK. In SFTS, elevation of CK levels is often observed, and CK is thought to be from skeletal muscle as a result of rhabdomyolysis or myositis [29]. Therefore, the source of elevated CK levels in the patient might be skeletal muscle rather than myocardium. Indeed, elevated troponin level in the absence of elevation of CK-MB level is not uncommon in myocarditis patients [30] but is uncommon in AMI patients. Additionally, her LV function returned to normal without coronary intervention. These findings suggest that her myocardial damage was due to myocarditis but not AMI.

**Table 1:** Clinical course of laboratory data

Days	1	2	3	4	5	6	7	8	9	10	13	14	20
White blood cell count (10^9^/L)	0.91	1.33	2.64	4.91	5.41	6.46	9.15	9.72	9.20	7.47	6.60	6.56	3.92
Neutrophils (%)	64	47	47			65.3	72.0			76.5	64.5		51.2
Lymphocytes (%)	28	46	37			21	23			13.5	27.5		39.0
Eosinophils (%)	0	0	0			0	0.1			0	0.5		0.3
Platelets (10^9^/L)	6.3	6.1	4.9	4.4	5.6	9.1	18.2	25.4	30.4	30.7	28.5	27.4	24.0
CK (IU/L)	317	314	213	351	648	1207	2484	1584	649	309	101	71	30
CK-MB (IU/L)	< 4	< 4	5	6	11	21	33	15	7		< 4		
AST (IU/L)	68		57	37	34	54	104	72	43	31	29	28	16
LDH (IU/L)	364		357	318	270	324	327	334	287	277	266	255	215
CRP (mg/dL)	< 0.02	< 0.02	< 0.02	< 0.02	0.03	0.03	0.03	0.05	0.07	0.10	0.03	< 0.02	< 0.02
Troponin I (pg/dL)			120.9		156.4		422.8		235.6		80.5		
Ferritin (ng/mL)	1947		2288			393	382	249	223	186	173		130

LDH: lactate dehydrogenase

We also suspected other etiologies that may have caused myocarditis. We performed virus isolation by a nasal swab; however, other viruses were not isolated. It was unlikely that she had bacterial or fungal myocarditis because CRP and culture results were always negative during hospitalization. It was also unlikely that she had eosinophilic myocarditis because there was no elevation of eosinophils levels during hospitalization (Table 1) and no cardiac symptoms, such as chest pain, dyspnea, and palpitations. On the basis of these findings, we diagnosed the patient as having fulminant myocarditis caused by SFTS without performing CAG and endomyocardial biopsy. However, the reason why this patient, unlike many other SFTS patients, suffered from fulminant myocarditis was not clear.

Several studies demonstrated that the disease severity of SFTS was associated with cytokine storm [31, 32]. In severe SFTS cases, cytokine storm often leads to fatal distributive shock, which is refractory to massive fluid resuscitation and maximum doses of vasopressor agents. In contrast, in this case, the patient needed not much fluid resuscitation and vasopressor agents but instead sufficient inotropic agents. Although the water balance of the patient had leaned toward excess for few days owing to cardiac tamponade and decrease in urine output (Fig. 3), the main cause of circulatory collapse in this case seemed to be cardiogenic rather than distributive. If we had continued massive fluid resuscitation in the way that we do for typical severe SFTS cases, the patient probably had developed heart failure and then would have needed some mechanical circulatory support.

Aynur et al. reported that severe and fatal Crimean-Congo hemorrhagic fever (CCHF), which has many similarities with SFTS (Bunyaviridae family, tick-borne infection, VHF disease, and other clinical findings [33]), was associated with impaired cardiac functions, such as EF depression, abnormal diastolic function, and pericardial effusion. This suggests that cardiac dysfunction may affect the prognosis of CCHF patients [34]. If the same is true for patients with SFTS, myocarditis may have been undiagnosed in previous severe SFTS cases.

We report the first case of fulminant myocarditis with SFTS. When treating SFTS patients who have experienced circulatory collapse, physicians should be aware of the possibility of fulminant myocarditis and perform echocardiography. When myocarditis is suspected, it is important to evaluate proper intravascular volume status and avoid indiscriminate, massive fluid resuscitation, and instead to administer sufficient inotropic agents. In the future, endomyocardial biopsy may provide additional insight into the pathogenesis of myocarditis caused by SFTS.

## Abbreviations

AMI: Acute myocardial infarction
AST: Aspartate aminotransferase
CAG: Coronary angiography
CCHF: Crimean-Congo hemorrhagic fever
CK: Creatine kinase
CK-MB: Creatine kinase-MB
CRP: C-reactive protein
ECG: Electrocardiography
EF: Ejection fraction
LV: Left ventricular
RNA: Ribonucleic acid
RT-PCR: Real-time polymerase chain reaction
SFTS: Severe fever with thrombocytopenia syndrome
VHF: Viral hemorrhagic fever
